# 10 recommendations for strengthening citizen science for improved societal and ecological outcomes: A co-produced analysis of challenges and opportunities in the 21^st^ century

**DOI:** 10.1371/journal.pone.0331161

**Published:** 2026-07-01

**Authors:** Jack S Nunn, Håkon da Silva Hyldmo, Lauren McKnight, Heather McCulloch, Jennifer Lavers, Julie Old, Laura Smith, Nicola Grobler, Cheryl Tan Kay Yin, Wing Yan Chan, Candice Raeburn, Nittya S. M. Simard, Adam Kingsley Smith, Sam Van Holsbeeck, Eleanor Drinkwater, Kit Prendergast, Emma Burrows, Christopher L. Lawson

**Affiliations:** 1 Science for All, Australia; 2 Department of Geography and Social Anthropology, Norwegian University of Science and Technology, Trondheim, Norway; 3 Faculty of Science, University of New South Wales, Sydney, Australia; 4 JBI, School of Public Health, University of Adelaide, Adelaide, Australia; 5 Adrift Lab, Underwood, Lutruwita/Tasmania, Australia; 6 School of Science, Western Sydney University, Penrith, Australia; 7 School of the Environment, The University of Queensland, St Lucia, Australia; 8 Queensland Trust for Nature, Queensland, Australia; 9 Department of Biochemistry and Pharmacology, Institute of Molecular Science and Biotechnology, the University of Melbourne, Melbourne, Australia; 10 Australian Institute of Marine Science, Townsville, Queensland, Australia; 11 Sunshine Coast Council, Caloundra, Queensland, Australia; 12 Reef Ecologic, Australia; 13 Forest Research Institute, University of the Sunshine Coast, Sippy Downs, Queensland, Australia; 14 Centre for Sustainable Agricultural Systems, University of Southern Queensland, Australia; 15 Florey Institute of Neuroscience and Mental Health, Melbourne, Australia; 16 Centre for Biodiversity and Conservation Science, School of the Environment, University of Queensland, Brisbane, Australia; International Islamic University Malaysia, MALAYSIA

## Abstract

Citizen science plays an increasingly important role in generating scientific knowledge and supporting environmental and social action. However, its potential to address complex global challenges remains underutilised. This study explores how citizen science can be improved by involving the public in all stages of scientific research. Using participatory research methods, online surveys and group discussions were conducted with researchers, citizen scientists, and Indigenous participants. Thematic coding was used to identify key challenges, opportunities, and best practices to enhance citizen science initiatives. Additionally, nine case studies were reported using the Standardised Data on Initiatives (STARDIT) reporting tool. The study identified key strategies for improving involvement, engagement and retention in citizen science initiatives. Findings underscore the importance of inclusive, evidence-informed approaches such as targeted outreach, fair compensation, tailored support, and co-creation practices. Ensuring data quality and fostering trust require adherence to FAIR data principles (findable, accessible, interoperable and reusable), transparent validation and sharing processes, and establishing ethical research partnerships. Persistent challenges include short-term funding, which undermines long-term project sustainability, and the lack of centralised support for ethics and project management. Formal recognition of citizen scientists through co-authorship, standardised training, and professional development opportunities can further strengthen involvement and build capacity. Finally, emerging technologies, including artificial intelligence and open data platforms, present opportunities to scale and improve efficiency, provided they are implemented with appropriate ethical safeguards and investment. Drawing together these insights, we provide 10 actionable recommendations for citizen science in the 21^st^ century. These highlight the importance of embedding citizen science in national research infrastructure, education, and policy, alongside consistent evaluation and reporting, to improve its inclusivity, longevity, and impact. We conclude by arguing that as the world confronts climate change, public health crises, and biodiversity loss, broader public involvement in science is key for equitable, efficient and evidence-informed responses.

## Introduction

Throughout history, knowledge-sharing has been a participatory process. The Universal Declaration of Human Rights recognises this by stating that everyone has the right to “receive and impart information and ideas through any media and regardless of frontiers” [1]. However, the past century has seen an increased division between those paid to conduct science, and those outside of academia or working without pay, including citizen scientists [2–4]. Citizen science is a flexible concept, but is guided by clear principles and values. Recognising that both the concept and terminology of citizen science are complex and flexible, this paper defines ‘citizen science’ as “the active involvement of the public at any stage of scientific research, with the aim to increase scientific knowledge, including by using the scientific method” [5].

Citizen science often emphasises partnerships among the public, non-professional scientists and professional scientists. These partnerships aim to democratise the scientific process by making it more inclusive, accessible and impactful. However, the concept of citizen science is not restricted to questions about who is ‘a professional’, who is being paid and who is not, nor is it limited to the data collection stage of research. Citizen science represents an open approach to science, which enables people to be involved in all stages of the scientific method, including identifying gaps in our knowledge and research priorities, developing research questions, designing methods, gathering and analysing data, evaluating research, communicating results, and implementing learnings [4].

Citizen science and its associated methods are not limited to certain academic disciplines, with fields as diverse as health, environmental and educational sciences. Across these disciplines, varied language is used to describe the same concepts of sharing power and responsibility with the public, and the democratisation of all stages of research [6]. While concepts of public involvement in health research and healthcare have been well-defined for several decades [7], more precise definitions and methodologies are still being discussed and refined across diverse disciplines [8,9]. This includes health research [10] and the ‘one health’ approach [11]; environmental research [12,13]; evidence synthesis [14,15]; urban planning [16]; education [17]; law [18]; policy [19,20]; and economics and budgeting [21]. Across these disciplines, it is useful to distinguish between scientific knowledge (what is known), the scientific method (how questions are answered), and the specific tools used to apply the scientific method. Many citizen science tools can be applied across disciplines.

Current philosophies of citizen science build on a range of related concepts [9,22]. One core concept of citizen science is ‘critical pedagogy’, which was defined by Paulo Freire as seeing “the world not as a static reality, but as a reality in process, in transformation” [23]. This inspired the development of ‘participatory action research’ as an approach to research methods where “communities of inquiry and action evolve and address questions and issues that are significant for those who participate as co-researchers” [24]. Other key concepts are ‘open science’ and the ‘open knowledge’ movement [25]. This is reflected in the United Nations Educational, Scientific and Cultural Organization (UNESCO) ‘Recommendation on Open Science’, which recognises that “science is a global public good that belongs to all of humanity” and aims to open “the processes of scientific knowledge creation, evaluation and communication to societal actors beyond the traditional scientific community” [26], making science “more accessible, inclusive and transparent” so that everyone can “share in scientific advancement and its benefits” [27]. Similarly, the World Health Organisation identifies the need for “a meaningful whole-of-society approach and social participation” to address issues of universal health coverage, and health and well-being [28].

### Motivation for this discussion paper

The benefits of citizen science are diverse and wide ranging, and can improve how we conduct research and understand our universe. Citizen science can strengthen public involvement and scientific literacy and improve collaboration between professional scientists and the public, as well as empowering communities at greater risk of exploitation, inspiring people to study and work in science, and building public trust in science through transparency and inclusivity [29–33]. It can also improve large-scale data collection (such as improving biodiversity monitoring), foster environmental stewardship, and support improved early threat detection in various disciplines.

Creating enabling conditions for inclusive citizen science can be crucial to project success. These conditions may be developed by building people’s capacity through training and development, developing institutional partnerships, and providing infrastructure, long-term funding, and accessible technologies that facilitate data collection and analysis [9,34,35]. However, initiating and sustaining citizen science projects with inclusive practices presents a range of challenges, including ensuring data quality, retaining participants, and addressing ethical concerns like consent and data ownership [36]. Additional challenges relate to inclusivity, communication, funding, and balancing scientific rigor with meaningful public involvement [37–39].

Recognising both these opportunities and barriers, this article aims to identify key themes, challenges and solutions for advancing and improving citizen science for all involved. To do so, we used a participatory action research approach to gather perspectives and experiences from experts, practitioners in the field and the wider public. This process enabled a joint exploration of how citizen science can be strengthened to address the societal and ecological challenges of the 21st century.

## Methods

### Methodological framework

This study uses participatory action research design and mixed-methods analysis. Participatory action research involves multiple stakeholders in the development, implementation, and evaluation of research [40–42], aligning the research process with the substantive focus of this study, namely shared governance and co-production in citizen science. We use the term ‘stakeholder’ here to include people with personal, professional or financial interests, as well as ‘interest-holders’ such as patients and people affected by enviromental pollution and climate change [43]. The study was co-created with citizen science experts from health, environmental, and education sectors across multiple countries. Participants collaboratively designed the survey, contributed to data analysis, and co-authored the resulting recommendations. The mixed-methods approach integrated inductive thematic analysis, computational text analysis and cross-case synthesis of illustrative case studies to enable triangulation and strengthen reliability and validity [44]. We transparently document the distribution of tasks among co-authors in the Methods and a Standardised Data on Initiatives (STARDIT) report, which is an open-access data-sharing system for standardising how information about initiatives is reported [45]. The Australian Citizen Science Association endorses STARDIT as a recommended reporting tool for citizen science initiatives, supporting consistent, transparent, open access and cross-disciplinary documentation of participation, process and impact.

### Data collection

#### Design and sampling strategy.

Two online surveys were used to collect data, with the results and participant feedback from the first survey refining the research scope and design of the second survey in accordance with participatory action research principles [42]. Round One of the survey was co-designed by the project coordinators, with the purpose and content of the questions discussed and refined with the lead author (JSN) and members of the Australian Citizen Science Association. Both surveys included an open invitation for co-authorship in the survey (following guidelines of the Committee on Publication Ethics (COPE)) [46]. Contributions by co-authors were transparently reported using STARDIT. Co-authors were invited to comment on the data, analysis methods, and to identify any perceived gaps in the data collected.

To identify barriers and opportunities for citizen science, the study used a combination of non-stratified venue-based purposive sampling and voluntary response sampling [47,48], enabling mobilisation of experiences, advice and participation from experts and practitioners with direct experience and knowledge about citizen science. Two main recruitment strategies were used for both surveys. Firstly, the surveys were circulated in established and relevant communities of practice through newsletters and online group postings. Secondly, and to identify experts and practitioners outside the identified communities of practice, the survey was posted openly on a range of social media platforms (see next section).

#### Online surveys.

A structured questionnaire was created using an online survey platform (Google Forms). The survey collected demographic data and featured open-ended questions about definitions of citizen science; areas of research or practice where citizen science could be better harnessed; examples of best practice (including an option to share URLs); challenges, opportunities and barriers faced by citizen science projects; and areas for growth and improvement (Appendix 1). The survey also asked respondents if they would like to contribute as co-authors.

The Round One survey was shared on 22 July 2024 and was open to responses for 67 days. Following the above sampling strategy, the Australian Citizen Science Association and Science for All shared the survey link via email newsletters and on multiple social media platforms, including Facebook, X (formerly Twitter), and LinkedIn. Co-authors identified in the Round One survey were invited to an online ‘kick-off’ meeting. Responses from Round One were analysed collaboratively to improve and refine data collection for Round Two. The wording of some questions was changed, and further questions were added to Round Two, which was shared using the same methods as Round One on 10 October 2024 for 21 days (Appendix 2). A final survey about people’s experiences of being involved in the project and using STARDIT was collected from 7–30 July 2025 (Appendix 3). Additional ideas that the group felt were important to the wider discussion were also added after the final data collection.

#### Collaborative process and documentation.

**Fig 1:**
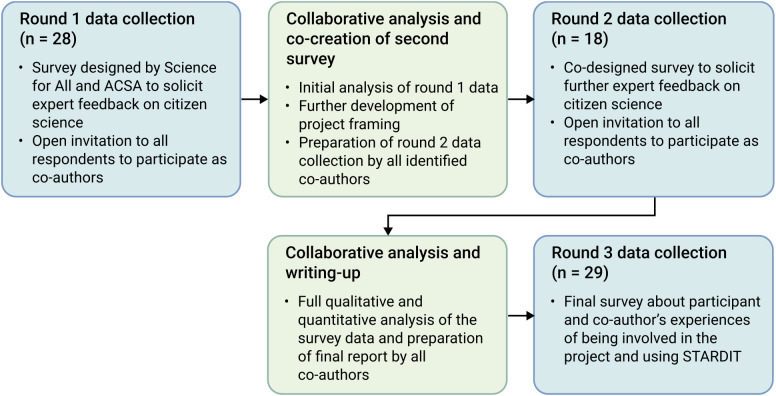
Schematic overview of co-production process. Overview of the co-production process used in the production of this paper.

The co-creation process invited people to volunteer their time, with the incentive of being a co-author on a peer-reviewed publication. Everyone who responded to the survey was invited to be a co-author at multiple stages throughout the process, including data analysis and write up. The co-production process involved 20 online meetings, shared collaborative documents and text-based discussion platforms to facilitate distributed participation across time zones. A record of all drafts, coding templates, meeting notes and analytic decisions was maintained in shared documents to create a transparent record of revisions and interpretations. These collaborative processes enabled iterative refinement of survey instruments, codebooks, and recommendations, while maintaining transparency in decision-making. The various tasks of individual contributions are documented in the associated STARDIT report. Co-authors agreed during the co-creation process that some people should be paid for some tasks. An overview of the co-production process is provided below (Fig 1). Further information is provided in the Supplementary Information and the STARDIT report about this article [49].

### Data analysis

#### Qualitative analysis.

We adopted an inductive thematic analysis approach consistent with participatory action research principles. An inductive approach was selected to allow themes to emerge from participants’ perspectives rather than imposing pre-existing theoretical frameworks. A multi-stage coding process was used to enhance analytical transparency, distribute interpretive authority among co-authors, and strengthen analytical credibility. The qualitative analysis was conducted in six stages by a sub-group of co-authors.

#### Stage 1: Dataset preparation.

Personally identifiable information, including names, contact details, and workplace references was removed. Each question was given an ID, and each survey respondent was assigned a unique identifier to allow re-identification if necessary. A master redacted dataset was created and securely shared with co-authors.

#### Stage 2: Inductive code creation.

Based on survey responses, the sub-group of authors jointly developed a draft set of codes based on a preliminary analysis of the submitted responses to capture key themes in the material. ‘Coding’ here refers to identifying and labelling key ideas or themes emerging from the data in a systematic way [48]. This initial set of codes was tested against survey responses, and an “Other – self-describe” category was added for flexibility. The coding system was refined through an iterative process that included online meetings with joint review of codes and survey responses that served as coder training and ensured completeness and usability for survey responses. The final draft of the codes was shared for analysis to categorise each response as either a ‘challenge’ or ‘opportunity’ within one or more themes.

#### Stage 3: Codifying responses.

Survey responses were randomly assigned to individuals for coding. Each team member applied relevant themes and sub-themes to their allocated data subset. Potential conflicts of interest were identified and addressed by reallocating response data. A live-shared analysis template, created with a shared online document, allowed identifying and adding new themes under the “Other” category as needed and discussing questions and challenging cases, both of which contribute to strengthening intercoder coherence [50,51].

#### Stage 4: Collating and refining codified responses.

All coded responses were consolidated into a single document and the code set was refined with the new themes and sub-themes identified in stage 3. A shared document was maintained for collaboration and running dialogue during the work.

#### Stage 5: Final coding and quality assurance.

A final round of coding was conducted using the updated code set. All text segments were coded by two co-authors. In addition, 20% of responses were coded by the lead author (JSN). To ensure consistent coding across the whole material, any discrepancies in coding were identified and resolved through joint discussions with all coders to reach intercoder agreement as a group.

#### Stage 6: Preparation for write-up.

The coded themes were converted into prose for the Supporting Information and structured into readable sections for the main article. The 10 Principles of Citizen Science as described by the European Citizen Science Association [52] and the Australian Citizen Science Association [5] were then mapped to the relevant themes. Based on these themes, the co-authors developed 10 recommendations to guide action in citizen science initiatives and programs, each directly responding to the key themes emerging from the data.

#### Quantitative analysis.

Quantitative text analysis complemented the inductive thematic coding by identifying lexical patterns across responses, allowing triangulation between manual coding and computational modelling [53]. Quantitative analysis was used to identify and count keywords in each theme that were identified by the qualitative analysis. We analysed responses regarding various aspects of citizen science from the two rounds of surveys. For open-ended questions, text analysis was performed using Python’s Natural Language Toolkit (NLTK). Responses underwent preprocessing, including lowercase conversion, special character removal, and tokenisation. To explore latent topic structures and assess the robustness of manually derived themes, modelling was implemented using Latent Dirichlet Allocation (LDA) and **scikit-learn**’s CountVectorizer for text vectorization. Custom stop words were employed and domain-specific stop words removed to improve theme coherence and analysis quality. Word frequency analysis was conducted using NLTK’s FreqDist. Data visualisation was performed using matplotlib and seaborn libraries, which generated response rate charts and word frequency distributions.

For Round 2, additional task-specific analyses were visualised using individual bar charts. The analysis was conducted using Python 3.13 with key libraries including NLTK, scikit-learn, pandas, matplotlib, and seaborn. All analyses were performed in a Jupyter notebook environment to ensure reproducibility and documentation. The combination of these methods provided a comprehensive understanding of patterns and trends in participant responses across both survey rounds.

For the final survey on people’s experience of being involved in this paper, we calculated the percentage of each response option in each question.

#### Ensuring reliability and validity through co-design and co-production.

Reliability was strengthened through co-designed methodologies, multi-analyst coding, peer validation of a subset of responses, and a final quality audit of 20% of the dataset. Validity was enhanced through methodological triangulation (manual coding, computational text analysis, and cross-case synthesis), transparent documentation of coding decisions, and participant validation of case study reports. The co-production approach distributed interpretive authority and reduced reliance on a single analyst, mitigating individual bias [50,51,53].

### Case studies and data reporting

Survey respondents provided examples of citizen science initiatives that exhibited best practices to benefit society. The co-authors conducted desktop research to gather supplementary publicly accessible information and data on these best-practice examples.

After analysis of the emerging themes from the qualitative data, the co-authors collaboratively selected citizen science initiatives that captured the breadth and variety in the material or were illustrative of certain themes. Case studies were purposively selected to reflect variation across geographic scale, disciplinary focus, technological integration, funding model, and level of participant involvement. Selection aimed to illustrate thematic breadth rather than statistical representativeness. We have included these illustrative case studies to demonstrate with real-world examples some of the themes we identified from the survey data [25]. The initiatives were analysed in more detail by extracting and structuring data about them using STARDIT data fields [45] (including aims, outcomes, data sharing and budgets), leading to the creation of publicly available STARDIT reports for each case study.

This entire process was conducted collaboratively and transparently, with co-authors verifying data accuracy. Organisations and individuals directly involved in each initiative were contacted via email and given the opportunity to review, amend, or improve or validate the respective STARDIT reports.

### Integration of participant feedback

Consistent with the participatory action research method, feedback from participants directly shaped the evolution of the study. Between Round One and Round Two, survey questions were refined to clarify terminology and expand the inquiry into payment structures and task distribution. During qualitative coding, themes initially classified under “Other” were incorporated into the formal codebook following co-author discussion, altering the final thematic structure. Draft recommendations were iteratively revised in response to co-author critique to strengthen clarity around equity, funding sustainability, and data governance. Case studies were also added or refined based on participant suggestions and validation feedback from people directly involved with the initiatives. This iterative feedback process ensured that both analytic framing and final recommendations reflected collective deliberation rather than a single-author interpretation.

### Ethical considerations for a co-created paper

Ethical considerations were embedded throughout the project design and implementation to support equitable participation, appropriate data stewardship, and transparent authorship. Indigenous participation was actively sought via the direct contact networks of project co-authors, funders, and collaborators, and through the broader newsletter and social media distribution channels described earlier in the manuscript, with the aim of reducing gatekeeping and ensuring opportunities to contribute were not limited to academic or institutional networks. How this project aligns with the CARE Principles for Indigenous Data Governance (collective benefit, authority to control, responsibility, ethics) [54] is reported in the associated STARDIT report [49].

All participants consented to the use of their survey responses for the purposes of this research. To protect confidentiality and minimise re-identification risk, names and other direct identifiers were redacted prior to analysis and reporting [55]. In relation to data ownership and use, participants’ contributions were treated as shared research inputs provided under explicit consent, with analysis and synthesis undertaken in a manner consistent with the project’s co-creation intent and with care taken not to attribute specific views to identifiable individuals or communities. Finally, to safeguard equity in recognition and decision-making, co-authorship was openly available to all participants in accordance with Committee on Publication Ethics (COPE) guidance, with an emphasis on transparent criteria and an inclusive pathway for contributors to opt into authorship and review how their contributions were represented. The study received ethics approval from the Norwegian Agency for Shared Services in Education and Research (reference number 107069). All participants provided written consent to participate in the project.

## Results

### Demographic data

46 respondents participated in the survey (28 in Round One, 18 in Round Two), sharing a total of 16,247 words in open text fields, with an average of approximately 350 words per respondent. Most respondents lived in Australia (80%), two (4%) of whom were Aboriginal or Torres Strait Islander. The majority of respondents considered themselves to be ‘academic’ and ‘have experience with academic writing’ (76%), including professional researchers paid to do research (65%). Most respondents also reported that they had experience as a volunteer researcher contributing to citizen science (65%). An overview of the demographics and previous expertise of participants is provided in S1 Table in the supplementary materials. All 46 respondents provided an email address and 34 answered that they wanted to contribute as a co-author. A total of 29 people completed the final feedback on the experience of being involved. A total of 18 respondents co-authored the final version of this paper (in line with the Committee on Publication Ethics Authorship guidelines [46]), and 6 asked to be acknowledged for their support. In total, people estimated that they volunteered 1,385 hours on this project.

### Data on payment for expertise in citizen science projects

**Fig 2:**
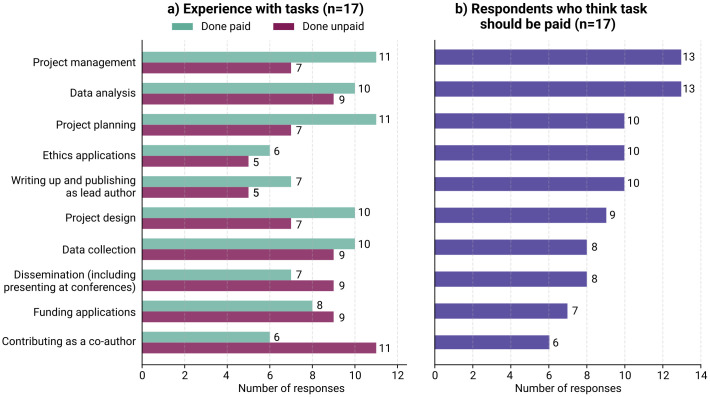
Results from Round Two survey on respondents’ experience and opinions regarding paid tasks in citizen science projects. **(a)** Tasks previously performed by respondents in citizen science projects, indicating whether they were undertaken as paid or unpaid work (multiple responses allowed per task). **(b)** Respondents’ opinions on which tasks in citizen science projects should be financially compensated, reflecting perceptions of task value, effort, or required expertise.

Round Two gathered data on tasks in citizen science projects, and 17 out of 18 respondents provided answers about whether they have completed paid or unpaid work in citizen science, as well as which tasks they think should be paid (Fig 2a). Most participants had experience contributing to academic publications, dissemination including conference presentations, and funding applications, and these tasks were predominantly performed without payment (Fig 2a). The remaining seven tasks were more commonly paid for, with the highest proportion of paid positions observed in project management, planning and design (Fig 2a). When asked about which tasks should be paid for, respondents showed the strongest support for data analysis, project management, and ethics applications (Fig 2b). While data collection was one of the most commonly performed paid and unpaid activities, there was relatively less support for making this a paid task (Fig 2b).

### Main themes from data

Eleven main themes with at least 15 responses per theme were identified through manual coding:

- **Recruitment and awareness (43 responses)**
- **Data collection and confidence (38 responses)**
- **Project support and capacity (26 responses)**
- **Attitudes (25 responses)**
- **Involvement and retention (24 responses)**
- **Individual capability and training (23 responses)**
- **Funding (23 responses)**
- **Inclusion and access (20 responses)**
- **Recognition of volunteers (20 responses)**
- **Technology and innovation (16 responses)**
- **Educational institutions (15 responses)**

### Overlaps and interplays in identified themes

There were considerable overlaps and interconnections between challenges and opportunities among identified themes. For example, attitudes towards citizen science was developed as its own theme, but was also indirectly related to funding, as public perception of citizen science was reported to affect its funding. Similarly, participant retention, data continuity and university engagement were all bilaterally related to funding, project capacity, and support.

‘Knowledge sharing’ was not classified as an individual theme but was prominent across many other themes. For example, there were calls for better reporting and knowledge sharing to improve citizen science awareness, volunteer retention, confidence in the data, attitudes towards citizen science, and collaboration with educational institutions. Similarly, ‘open data’ appeared in five responses directly and four others indirectly. For example, under ‘participant acknowledgement’, responses noted that participants should have access to their own data to ensure the program is ethical and to improve retention. Related, a lack of confidence in data was the second most common theme and influenced other themes including attitudes towards citizen science, funding acquisition, technology, and volunteer retention.

**Fig 3:**
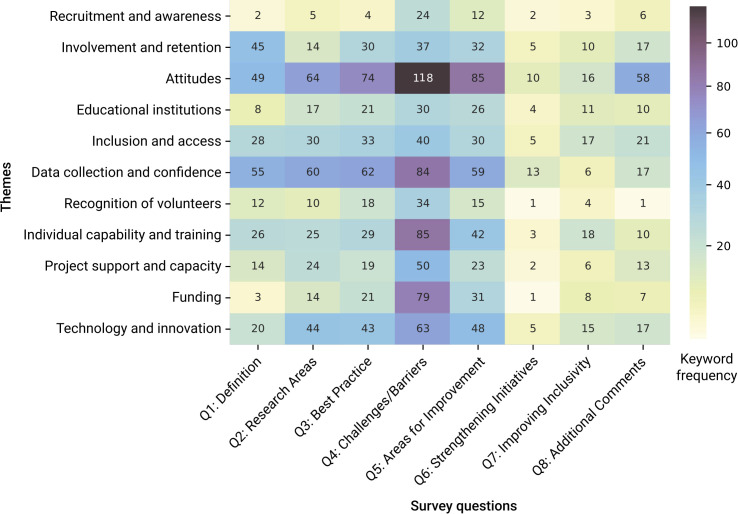
Heatmap visualisation of the frequency of theme-related keywords in participant responses. The colour intensity and numerical values represent the prevalence of each thematic area, revealing which themes dominated specific questions and identifying patterns in participant discourse across the survey. The full list of keywords associated with each theme is provided in S2 Table in the supplementary materials.

An overview of the most prominent themes, including ‘attitudes’, ‘data collection’ and ‘confidence’ that arose consistently in responses to most survey questions is provided in the heatmap visualisation in Fig 3 below.

An overview of the themes, challenges and opportunities with illustrative quotes identified through the analysis, as well as how they align with the 10 Principles of Citizen Science [5], is included in Table 1 below.

**Table 1:** Summary of themes, challenges and opportunities. Abbreviations: CS = Citizen science; EMCR = early-mid career researcher.

Themes (number of responses)	Challenges	Opportunities	Illustrative quotes	Alignment with 10 Principles of Citizen Science
**Recruitment and awareness [43]**	1. Recruitment of new participants into CS projects. 2. Lack of public awareness of projects and how to become involved. 3. Lack of support and resources available for researchers recruiting volunteers. 4. Perceived inaccessibility to CS projects by non-scientists.	1. Employ marketing and science communication strategies to increase awareness. 2. Target schools for recruitment. 3. Communicate benefits of participation and consider the needs of participants. 4. Providing tools to connect volunteers to projects. 5. Build relationships and collaborations for meaningful engagement.	“There is a lack of infrastructure to support making it easier for EMCRs and community to connect” [R15] “People hear ‘science’ and think if they are not a scientist they can’t get involved.” [R34] “Excellent support from marketing experts and teachers to help attract and retain participants really effectively” [R13]	P1 - active involvement.
**Data collection and confidence [38]**	1. Widespread concerns of poor data quality or perception of poor data quality (28 responses). 2. Poor data continuity due to volunteer retention, funding, and unreliable long-term expert collaboration, for example university researchers on short-term contracts.	1. Robust data validation, including standardised training, quality control, and data management. 2. Ensure data is shared according to FAIR principles [56]. 3. Use standardised ways of reporting methods (including data collection). 4. Communicate data validation work to enhance trust and usability. 5. Potential for scalable data collection if challenges are addressed.	“Provision of open-access databases, QC protocols, and appropriate metadata standards can help with data validation and reliability.” [R11]. “There is a genuine lack of support for citizen science at both state and commonwealth levels which means it isn’t considered a valid data source” [R40]. “There has to be a way to leverage the ability of citizen science to be almost everywhere at once to help inform research.” [R10].	P1 - active involvement. P2 - genuine outcome. P6 - controlled limitations. P10 - law and ethics.
**Project support and capacity [26]**	1. Lack of centralised support for operationalising robust CS projects, for example project management positions at universities or not-for-profit organisations. 2. Lack of electronic and online infrastructure that is easily usable by citizen scientists.	1. Provide centralised services to support project management, ethics and data management. 2. Develop institutional infrastructure that lowers the barriers for starting citizen science projects and supports project implementation and connection and engagement with communities. 3. Ensure the public are involved in all stages of the research, and there is transparent reporting about project capacity	“For some species-specific issues, there are no websites, databases or other resources available to promote and support data collection” [R14]. “A general hub with these [supportive] resources (national and international) would help find these resources” [R35]. “(Creating) enabling conditions for self-managed science” [R46].	P4 - multi-stage participation. P6 - controlled limitations. P10 - law and ethics.
**Attitudes [25]**	1. Perception of science as boring hinders citizen engagement. 2. Perception of CS as not robust. 3. Universities can become disinterested because of a lack of direct funding for CS activity. 4. Scientists and medical professionals may think CS is ‘outreach’, not research.	1. Increased and improved outreach and information informing citizens about aims and opportunities to be involved. 2. Government is interested in CS because it’s seen as inexpensive. 3. Create more consistent and clearer language to describe CS across disciplines. 4. Improve people’s attitudes by conducting research on community attitudes toward CS; improving quality of projects, making benefits of CS clearer; improving information dissemination; and improving industry alignment.	“Less authors lead to a more prestigious paper and higher recognition” [R43]. [In Australia] “in health and medical research, we talk more about ‘consumer engagement, consumer involvement and consumer led’ rather than citizen science. So, there are also language issues to contend with” [R15]. “Unless you work in science, most people have a feeling about science as something hard and boring and for geeks” [R15].	P2 - genuine outcome. P3 - benefits science & society. P8 - participants acknowledged. P9 - outcomes acknowledged.
**Involvement and retention [24]**	1. Retention of existing citizen scientists for involvement in ongoing data collection. 2. Delay between data collection and research outcomes hinders sustained involvement. 3. Lack of funding and resources for ongoing engagement. 4. Reduction in data quality with loss of CS participants.	1. Design projects that are well-organised to facilitate participation. 2. Employ systems and personnel to manage volunteers. 3. Identify, provide and communicate benefits to participants. 4. Provide agency to citizen scientists by giving ownership beyond data collection, and by collaborating with key stakeholder groups.	“When people are just getting into science they need a fairly fast turnaround time between data collection, analysis, and results. The long arc of a research project is difficult to stay engaged with when it’s just casual engagement.” [R39] “I think active participation is also important: moving beyond data collection to engage community members in multiple steps of the research” [R1] “Ensuring these [data collection] platforms are accessible to all skill levels and intuitive to use, would trigger ongoing participation of the public.” [R43]	P3 - benefits science & society. P4 - multi-stage participation. P5 - feedback provided.
**Individual capability and training [23]**	1. Researchers lack training to involve citizen scientists, identify appropriate collaborating organisations, and design projects. 2. Training of citizen scientists can be insufficient to meet program goals. 3. Meeting training needs requires expanded funding models and supports. 4. Researchers lack the time for high quality CS engagement.	1. University-run training for researchers on developing successful citizen science programs. 2. Community-driven training of researchers from experienced practitioners. 3. Formalised training standards for citizen scientists, including micro-credentials for complex topics which can have career benefits for volunteers. 4. Universities create capacity and recognise the time required to work with the public to create and sustain citizen science projects.	“Academics sometimes lack the time and the skills to engage with citizen scientists” [R21] “Researchers not knowing how to find citizen science groups, … engage with sometimes large groups of very diverse people, … and how to ensure integrity of data and analysis in a way that will stand up to the rigorous peer-review process.” [R38] “Universities could provide resources and training on stakeholder engagement or establishing collaborations.” [R21] “Appropriate training can enhance reliability and transparency of the data, as well as improve job prospects for participants.” [R11]	P2 - genuine outcome. P3 - benefits science & society. P6 - controlled limitations.
**Funding [23]**	1. General lack of direct funding for CS as a methodology across all fields. 2. Limits research partnerships: short-term grants cause problems for recruitment, training, and institutional development needed for engaging volunteers in long-term CS projects. 3. Affects data continuity.	1. Develop mutually beneficial, ethical and transparent partnerships with industry. 2. Include dedicated funding for CS in existing grant schemes, including grants that better fit the design of CS compared to traditional research projects.	“They put in a lot of effort and learn a lot, but then funding is done and people move on” [R39]. “Invasive species, loss of biodiversity, climate change, [and] bushfire risk do not have an end-date [like funding cycles do]” [R45]. “Harnessing the unique selling points of citizen science (...) to attract more funding” [R20].	P1 - active involvement. P2 - genuine outcome. P3 - benefits science & society. P9 - outcomes acknowledged.
**Inclusion and access [20]**	1. People involved in CS can be seen as simply data collectors without authentic involvement in the research process. 2. Ethical data sharing and intellectual property rights, especially when working with Indigenous participants.	1. Include citizen scientists in all stages of CS including identifying topics and priority setting to create better connections between the public and professional scientists. 2. CS can help the public be involved in science in a meaningful way, ensuring it aligns with the values and priorities of the public.	“I think one of the challenges is the ‘power’ structure of research, that doesn’t easily lend itself to ‘ideas/assistance from the uninitiated’ (…) scientists are put on a podium for the work they do, and there is not really knowledge of how non-scientists might contribute. We need to make it possible for there to be a ‘space for everyone’ in science, and obvious, accessible pathways into all those spaces” [R15]. “Creating guidelines that emphasize ethical data sharing, intellectual property rights, and co-design of research projects with Indigenous communities would promote more inclusive and respectful collaborations, ultimately benefiting both science and society” [R27].	P1 - active involvement. P4 - multi-stage participation. P7 - open data. P10 - law and ethics.
**Recognition of volunteers [20]**	1. Volunteers are under-acknowledged and require additional support or recognition to ensure they feel their contributions are valued. 2. Volunteers may not have access to the data they’ve contributed – implies an ethical issue of public data availability and impacts retention.	1. Allocating resources to provide support and recognition can encourage ongoing involvement. 2. Rewards, reimbursements and co-authorship can provide recognition. 3. Better support for intermediaries between citizen scientists and researchers to support volunteers, such as community organisations. 4. Citizen science volunteers can co-manage projects alongside researchers.	“Citizen scientists should be rewarded for their participation via certificates, newsletters, training” [R43]. “Volunteers need to have better access to the data they themselves collect, because it is THEIR data (normative reason), because they care about and for data and not having access is detrimental for volunteer retention (instrumental reason) and because granting access could allow volunteers to do more with data (such as education and advocacy outside of the citizen science project’s scope).” [R16] “I think a formal intermediary role which could work between the parties would be hugely beneficial. Again, this could involve different levels of service/support based on needs of the group, but the focus should always be on multidirectional value exchange (citizen scientists learning and acknowledged contributions, scientists gaining additional data and insights, broader community receiving value from new knowledge).” [R38]	P4 - multi-stage participation. P5 - feedback provided. P7 - open data. P8 - participants acknowledged. P10 - law and ethics.
**Technology and innovation [16]**	1. Redundant apps without innovation: many similar apps for CS can cause participants to be overwhelmed or bored. 2. Novel apps are technically difficult and expensive to build, with little support available. Difficult to keep up to date (software, security and interoperability). 3. It is difficult for some to find relevant CS apps based on their interests, despite there being many available.	1. Technology has a crucial role in enhancing data accuracy, scale, participation and accessibility, ranging from visually impaired people to remote communities. 2. Support from corporations or governments in digital innovation can improve user experience and data interoperability amongst programs. 3. Central hubs that provide resources to help build digital infrastructure for CS projects and for participants to find CS projects.	“Participants say “not another app” - [CS should] find innovative approaches that increase diverse participation, retention and enjoyment for all involved” [R29]. “There are already apps for almost everything you can think of nowadays, but nowhere to find an updated catalogue.” [R7]. “Once [citizen science] is accepted more we can rapidly expand the scope of work and possibility of what can be achieved, especially with the use of AI (Artificial Intelligence).” [R20] “More engaging, interactive mediums like apps that have dedicated teams updating and refining the front-end to ensure users can utilise the tools successfully.” [R7].	P2 - genuine outcome. P3 - benefits science & society.
**Educational institutions [15]**	1. Lack of awareness of the benefits of CS to school students and staff. 2. Difficulty maintaining continued student involvement in projects.	1. CS is an important tool for engaging and motivating students in science and solving real-world problems. 2. Strong partnerships with universities, including opportunities to build CS into curriculum and award certifications.	“School-based citizen science programs are a win-win (…) [schools] benefit from engaging with authentic scientific practices…” [R39]. “Appropriate integration of education [is important] (…) citizen scientists should be rewarded for their participation via certificates, newsletters, training…” [R43].	P1 - active involvement. P3 - benefits science & society.

### Illustrative case studies

Survey participants shared 45 citizen science initiatives and an additional four were later identified by co-authors. Seventeen STARDIT reports were created from the 49 examples, which were used to help select the final 9 illustrative case studies (Table 2). Of the 9 case studies, we invited people from all the initiatives to validate the data about them in the STARDIT reports, with 7 not replying, and 2 validating the data.

**Table 2:** Illustrative case studies.

Title	Locations and Dates	Description	Aims	Outputs and impacts	Illustrative of key themes	STARDIT report URL and ID
**Coral Watch** (coralwatch.org)	Over 80 countries, available in 12 languages. 2002 – present.	Monitors coral health using a simple Coral Health Chart to assess bleaching. Combines scientific data collection with education and outreach, making reef monitoring accessible to volunteers and students worldwide.	Raise public awareness of reef conservation, engage volunteers in monitoring coral health, and provide accessible scientific data on reef conditions. Promotes education and community action to address climate change and coral bleaching.	Hosts a publicly accessible coral bleaching dataset and an interactive data map, contributing data from over 2,362 reefs in 80 countries [57]. Engages the public through events, workshops, and ambassador programs, fostering community involvement in reef monitoring and conservation.	**Involvement and retention, Inclusion and access:** Simple and accessible, requiring minimal training and equipment. **Data collection and confidence:** While the calibrated colour chart enables high-quality science, human judgement is still necessary. Data reliability is supported by trained and paid ambassadors and staff. **Educational institutions:** strong ties to university researchers and admin support.	https://stardit.wikimedia.org.au/wiki/0202410211107
**Reef Check** (reefcheck.org, reefcheckaustralia.org)	Global, with country-specific branches, e.g., ‘Reef Check Australia’. 1996 – present.	Provides volunteers with structured training programs (e.g., line-transect survey) to help protect marine environments through citizen science and community engagement. Connects people with reef research and supports local conservation efforts and global participation in reef monitoring.	Build community capacity for collecting, interpreting, and acting on reef health data. To provide locally relevant, globally comparable data, fill gaps in monitoring, and inspire community-driven actions to protect reefs.	Conducted surveys in 102 countries. Provides data for reef management and, through partnerships with tourism operators, offers cost-effective surveys with ~93% accuracy [58].	**Individual capability and training, Data collection and confidence:** Volunteer training program is detailed (multi-day, in-person and in-water) and consistent among participants to strive for quality control. **Recruitment and awareness:** Partnerships with the tourism industry and volunteers enable cost-effective, scientifically validated data collection (~93% accuracy) through line-transect surveys. **Project support and capacity:** Global initiative with multiple branches.	https://stardit.wikimedia.org.au/wiki/0202410170606
**Great Reef Census** (greatreefcensus.org)	In-water: Great Barrier Reef (Australia, 2020 – present), with pilot studies globally starting 2025. Online analysis: Over 80 countries.	A collaborative initiative to survey the health of coral reefs, using AI, marine scientists, corporate technology and resources, and citizen scientists. Citizen scientists collect photos in-water and analyse them from anywhere globally. Data are provided to university scientists who advise reef managers to optimise locations for direct practical coral protection efforts.	Capture large-scale data that support research and management. Establish a scalable, bottom-up, decentralised and financially sustainable approach to conservation. To foster a sense of stewardship for the reef among participants and the wider public who otherwise wouldn’t be exposed to environmental issues.	Surveyed ~800 reefs in 5 years (~25% of the GBR, previously only ~5% was regularly surveyed). Over 12,000 global participants. Average cost to survey a reef significantly cheaper than existing methods and 99% accurate [59]. Data is used in research and provided to government, e.g., to guide pest management.	**Technology and innovation, Data collection and confidence, Funding:** Combines citizen science and AI to improve scale while maintaining accuracy and is supported through corporate partnerships to help data continuity (long-term funding) and digital innovation. **Recruitment and awareness, Involvement and retention, Inclusion and access, Educational institutions, Attitudes:** Brings world-class science to resource-poor communities, lowers participation barriers for diverse groups, and engages thousands of people globally, including school children and corporate workers, in environmental issues. **Recognition of volunteers:** Gives volunteers access to their own data and a dedicated not-for-profit organisation supports linking researchers and citizen scientists.	https://stardit.wikimedia.org.au/wiki/0202410282330
**Marine Debris Initiative** (tangaroablue.org/about-amdi)	Entire Australian coastline, including remote beaches, islands, and underwater locations. 2004 – present.	Monitoring and removal of marine debris by citizen scientists, including data recording of location and type of debris. Volunteer-driven project supported by 2,000 diverse partners and expert scientific advice.	Support marine conservation by removing and preventing marine debris, collecting data on pollution, and educating and engaging the community.	Collected over 20 million data points on marine debris that are available on a web-based visualisation tool for environmental research, management and science communication. Identified hotspots and major sources of pollution, including plans to reduce these sources. Contributed to policy changes such as single-use plastic bans. Contributed to multiple research projects and industry reports [60].	**Attitudes, Recognition of volunteers:** Helps inform policy. Gives volunteers access to their own data. **Data collection and confidence:** Communicates data validity, including measures of uncertainty. **Funding, Project support and capacity**: Government funding support enables ongoing monitoring and therefore data continuity.	https://stardit.wikimedia.org.au/wiki/0202410171055
**WomSAT** (WomSAT.org.au)	Multiple states and territories in Australia. 2015 – present.	Monitors and protects wombat populations through observational data collection and conservation efforts. Supported by universities, it engages citizen scientists in collecting research data, awareness campaigns, and training courses, including mange treatment.	Gather data on wombat populations and threats, support wombat conservation efforts and educate and engage the wider community.	Over 23,000 citizen science records have advanced research on wombat conservation, revealing patterns in sarcoptic mange and roadkill hotspots [61–63]. The data is accessible via an interactive online map. Successfully built a community that advocates for wombats.	**Individual capability and training, Recruitment and awareness:** Combines training, public engagement, and accessible data presentation. Offers citizen scientists robust training courses on wombat ecology and mange treatment. **Recognition of volunteers, Data collection and confidence:** The interactive website ensures data is easily accessible to the public, researchers, and policymakers.	https://stardit.wikimedia.org.au/wiki/0202410220432
**Galaxy Zoo** (zoo4.galaxyzoo.org)	Global. 2007 – present.	Designed to confirm the identity of galaxies through online imagery. The original project ended in 2009; however, it has been re-launched multiple times and includes Galaxy Zoo, Galaxy Zoo 2, Galaxy Zoo: Hubble and Galaxy Zoo: CANDELS. Multiple universities and astronomy institutions are involved.	Identify galaxies by confirming their spiral structure and determining their direction of rotation. Minimise telescope time spent on non-galactic observations.	In the first year 50,000,000 galaxies were classified by citizen scientists [64]. The project has enhanced understanding of the universe and resulted in over 650 publications and data releases.	**Inclusion and access, Data collection and confidence, Educational institutions**: Accessible participation in citizen science globally that directly contributes to research studies.	https://stardit.wikimedia.org.au/wiki/0202412120556
**Citizens for Refuge Ecology (C4RE) Camps**	12 individual camps across three Nature Refuges in SE Queensland, Australia, 2021–2023	Conducted biodiversity monitoring on private land by connecting landholders, species experts, and local communities in one-off overnight events. These citizen science camps focused on surveying mammals, plants, birds, and invertebrates, using platforms like iNaturalist and eBird to record data. Funded by the Office of the Queensland Chief Scientist.	Empower, upskill, and motivate attendees through learning survey techniques and connecting with nature. Learn more about the species on private landholders’ property. Facilitate species experts passing on their knowledge and build the capacity of the wider community.	All camps discovered previously unrecorded species at that location. New species were discovered, primarily invertebrates and gall-forming species. Extended population ranges were identified, and critically endangered species were recorded. In total, over 1,600 species recorded and over 3,600 observations made publicly available.	**Attitudes, Involvement and retention**: Participants recorded improved perception on their own agency to improve nature. **Individual capability and training, Data collection and confidence:** In-person training enabled replicable data (within camps and elsewhere). **Funding, Project support and capacity, Recruitment and awareness:** Highlighted the value of government funding commitment over multiple years. Also provided field training for research scientists, who in turn built community engagement with other participants.	https://stardit.wikimedia.org.au/wiki/0202410171213
**Sweaty and Stressed**	Australia-wide (all states and territories). Australian summer of 2022/23	Eighty renters across Australia tracked indoor temperatures using monitoring devices, generating a detailed dataset of temperature fluctuations. Surveys and interviews provided qualitative insights into renters’ experiences and the impact of indoor heat.	Gain insight into the experiences of renters who struggle to maintain a comfortable temperature in their homes.	Poor insulation and limited cooling options left renters in substandard housing exposed to unsafe indoor heat, impacting their well-being. Rising energy costs forced many to ration cooling or endure extreme temperatures, increasing energy debt and highlighting the need for better rental standards.	**Attitudes, Recognition of volunteers:** Citizen science that can contribute to research and public discussion on policy. **Inclusion and access, Technology and innovation, Data collection and confidence:** This was accessible and easy (minimal training or time commitment) to contribute, aligning with the scalability of citizen science, e.g., there are millions of renters across Australia so we can harness that resource to gather basic information that can help shape policy on public health, housing, economics and climate change.	https://stardit.wikimedia.org.au/wiki/0202502222026
**NOBURN**	Australia-wide. Launched 2021, operating across Australia since 2023.	Through the NOBURN app, citizen scientists upload photos and observations of forest fuel loads and management, assisting with hazard assessments to predict bushfires. AI is used to analyse images with support from university scientists.	Using AI, time-stamped and geo-referenced photos from the public are analysed to assess forest fuel structure, quantity, density, and dryness. It aims to improve predictions of fire likelihood, severity, and extent, enhancing Australia’s disaster resilience and preparedness.	Although in its early stages, the project hopes to improve information and decision-making for firefighting authorities.	**Technology and innovation, Data collection and confidence, Inclusion and access:** Utilises AI to reduce training requirements of participants and resource demands of image analysis.	https://stardit.wikimedia.org.au/wiki/0202410160223

### Cross-case analysis

A cross-case analysis of the nine illustrative case studies identified several key patterns in citizen science initiatives and provides examples of technologically integrated and collaborative approaches to enhance sustainability, data quality and public involvement. Projects varied in accessibility and citizen scientist commitment. For example, three projects focused on coral reefs but used different approaches. CoralWatch enables broad, low-barrier in-water monitoring; Reef Check requires extensive training to support data reliability; while the Great Reef Census enables involvement in reef conservation from anywhere globally.

The integration of artificial intelligence (AI), as demonstrated in the Great Reef Census and NOBURN, reduced reliance on expert training, and enabled broader involvement. The use of AI to analyse photos uploaded by citizen scientists also reduced manual data processing, minimising human error and improving data accuracy.

Many initiatives foster deeper community stewardship by emphasising individual involvement and education, such as WomSAT and Citizens for Refuge Ecology (C4RE) Camps. CoralWatch, WomSAT and the Marine Debris Initiative are examples of how interactive online platforms can be used to involve and educate volunteers and give them access to data they have collected. These platforms are presented in user-friendly and accessible formats, such as interactive maps.

Collaboration between paid scientists, unpaid volunteers, and partner organisations is highlighted as a key factor in project sustainability. However, funding models can differ. Corporate partnerships helped support the longevity and digital capabilities of the Great Reef Census, whereas C4RE Camps and other projects showcase the immediate benefit of short-term government grants. These variations highlight the evolving nature of citizen science, where balancing accessibility, data reliability, technological innovation, and funding stability remains a central challenge for long-term impact.

### Participation experience data

We collected data about people’s experience of being involved in this project, with 55% (15/27) responding ‘I felt I could be involved in all tasks of the project’, 45% stating ‘I felt I could be involved in some tasks’ and no one responding ‘I felt there was no opportunity to be involved in any tasks of the project’. When asked ‘Would you recommend using STARDIT to report citizen science or other initiatives?’, 10 people responded to the question, and 90% (9/10) responded yes, with one ‘maybe’.

## Discussion

The results demonstrate that the concept of ‘citizen science’ is transitioning from a peripheral data-collection activity to a central pillar of knowledge co-production. However, it remains constrained by structural inefficiencies and a lack of formal recognition. Our co-produced results identified key challenges for citizen science, including participant recruitment and retention, ensuring data quality and trust, short-term funding limitations, and lack of payment or formal recognition for contributors. These challenges did not emerge as isolated operational issues, but as structurally interconnected constraints. For example, funding instability directly degrades data continuity and participant retention. These findings align with prior research identifying structural precarity in citizen science funding models [3,6], but extend this work by empirically linking funding instability to data continuity and participant retention within a co-produced analytical framework. While earlier studies have framed citizen science primarily as a data-expansion strategy, our findings reinforce arguments that institutional design and recognition systems fundamentally shape scientific credibility and participant equity.

Effective strategies included transparent data practices aligned with FAIR data principles (findable, accessible, interoperable and reusable) [56], inclusive involvement methods, sustainable funding models, centralised support infrastructure, and expanded training and career pathways for participants. Case studies also highlighted the potential of technology and innovation. For example, data platforms and AI enhance scalability by reducing the manual burden and reducing barriers for the public to be involved (as seen in the Great Reef Census and NOBURN).

While citizen science is often framed as inherently democratising, our data suggest that without institutional reform it may reproduce or reinforce existing hierarchies, particularly in relation to authorship recognition, funding allocation, and the ‘trusted’ epistemic authorities. This highlights a tension between aspirational rhetoric and operational realities.

Here, we provide 10 actionable recommendations in response to emergent themes that citizen science initiative operators, funders, **advocates** and policymakers can enact to improve citizen science and increase its scope for benefit to society and the environment. These recommendations advance the field by shifting the focus from individual project survival to the creation of a robust, institutional ecosystem for participatory research. We also place our key themes and recommendations in the context of broader financial, ethical, political and legal issues. The findings advance the field of citizen science by demonstrating how methodological robustness, citizen scientist recognition, and institutional design jointly shape data credibility and sustained involvement. In doing so, the findings reinforce and extend existing literature that positions citizen science as a form of knowledge co-production which requires explicit structural support, rather than solely a data-collection method [3,6,8].

Beyond reinforcing existing scholarship, this study contributes three advances to the field. First, it empirically demonstrates how structural factors—particularly funding stability, recognition systems, and institutional infrastructure—shape both data credibility and participant retention. Second, it integrates operational challenges (such as training, data validation, technology) with governance-level design considerations, reframing citizen science as an institutional ecosystem rather than a collection of discrete projects. Third, by embedding co-production within the research process itself and reporting contributions, inputs and outcomes transparently through STARDIT, the study offers a replicable model for reflexive and accountable citizen science. Together, these contributions move the field from descriptive advocacy toward evidence-informed institutional design.

### Recommendations

- 1. **Strengthen institutional and government support, and develop publicly led ‘hubs’**: Developing infrastructure, funding mechanisms, and policy alignment is important for sustaining long-term citizen science programs [3,6,25]. These challenges could be addressed if governments provide long-term funding for ‘super hubs’ that support institutions to host multiple citizen science projects. These ‘super hubs’ would use centralised funding and infrastructure for administration, ethics, project management, data hosting and sharing, communications, reporting and evaluation, and address the lack of centralised support. For example, a ‘super hub’ at a university could build partnerships with schools, communities, specialist clubs (such as dive clubs and natural history societies) and beyond, while sharing control of the individual citizen science projects with the public. The proposed ‘super hubs’ model is not widely formalised in the literature, but it aligns with calls for shared infrastructure, coordination, and public governance [3,25,26].
- 2. **Improve partnerships with schools and educational institutions:** Findings under the emergent themes of ‘Educational institutions’ and ‘Recruitment and awareness’ highlighted low awareness of citizen science benefits and difficulties in sustaining student involvement. The WomSAT and CoralWatch case studies suggest that integrated education leads to higher engagement. Strengthening partnerships between schools and higher education institutes can increase the number of people involved and the exchange of skills and knowledge among students, teachers and researchers. This could be achieved by integrating citizen science into curricula and providing appropriate funding and resourcing, such as training for early- to mid-career researchers and education professionals on developing partnerships. Citizen science can be used as a tool for expanding science knowledge and scientific literacy [36], and enhancing public understanding of science [29].
- 3. **Improve communication about citizen science:** Evidence-informed communication methods should be used to inform the public and raise awareness about citizen science opportunities and their benefits. Increasing research on knowledge and attitudes toward citizen science is important to identify gaps, involve more people and work in ways that align with their needs and preferences. This addresses the finding in the ‘Attitudes’ theme that science is often perceived as ‘unengaging’. Targeted communication can address misperceptions that currently undermine recruitment, funding, and institutional legitimacy [3,65]. This recommendation draws on findings from ’Attitudes’, ‘Recruitment and awareness’, and ‘Involvement and retention’ themes.
- 4. **Improve citizen scientist support and recognition**: Multi-platform tools, training, and project management should be used to support inclusive ways of working and ensure data quality. Improved mechanisms to recognise citizen scientists are important and may include providing remuneration, clearer guidelines on acknowledgement and authorship in research articles, and better reporting on the contributions of citizen scientists using tools such as STARDIT [45]. This recommendation is informed by findings related to the themes ‘Recognition of volunteers’, ‘Funding’, and ‘Data collection and confidence’, which highlighted recurrent under-recognition of volunteer contributions. These findings identified links between recognition, participant retention, and the continuity and reliability of collected data. They are consistent with established principles of citizen science that emphasise participant acknowledgement, feedback and ethical practice as core elements of effective participation [5,52]. These findings support existing literature showing that participant motivation and sustained engagement are influenced by how volunteer contributions are recognised and supported within projects [7,39].
- 5. **Improve transparency about methodology and processes**: Developing standardised frameworks for citizen science projects is key, including planning, reporting and evaluation. Quality assurance processes should be linked to reporting standards. Information from these frameworks can improve data validation, quality control, and communication about data reliability. Case studies using such frameworks (e.g., Reef Check’s training-intensive model) can help demonstrate the value and effectiveness of citizen science methods, and any associated participatory methods.

Data about citizen science, or collected and analysed through it, also needs to be publicly accessible to facilitate validation of its accuracy and/or reliability, as a perception of low-quality data can hinder data usage, future funding, and participant retention. Project design should include funding for scientific expertise to address concerns about data quality, help develop robust protocols, and to validate data. This means involving scientists from the outset, rather than having organisations design citizen science projects in isolation. Improved transparency about how data was collected and analysed can help improve trust in citizen science and improve data reliability and usability. This recommendation results from findings under the theme ‘Data collection and confidence’ and ‘Attitudes’, where concerns about perceived data quality repeatedly influenced funding, uptake and legitimacy. Transparent methodological reporting addresses both real and perceived limitations. This strengthens the evidentiary standard of citizen science in line with FAIR and open science principles [56].

- 6. **Follow FAIR data principles**: Clear reporting standards enabling FAIR data (findable, accessible, interoperable, and reusable) remain critical for impactful citizen science [56]. FAIR-aligned data practices mitigate ethical risks, improve re-use, and maximise long-term impact beyond the lifespan of individual projects [56]. Following FAIR data principles requires building, hosting and maintaining digital infrastructure for collaboration, including working with trusted partner organisations such as the Wikimedia Foundation and affiliated national chapters such as Wikimedia Australia. Large open data aggregators for citizen science should be utilised wherever possible, such as the Global Biodiversity Information Facility. All government-funded research should mandate FAIR data sharing.
- Such data sharing should be mindful of the United Nations Declaration on the Rights of Indigenous Peoples (UNDRIP) which declares the rights of Indigenous peoples ‘to maintain, control, protect and develop their intellectual property’. The framing of knowledge as “data” to be owned, shared, or made “open” can itself reflect colonial logics that extract and commodify Indigenous knowledge outside of its cultural and relational context. The CARE Principles for Indigenous Data Governance [54] provide a complementary framework recognising Indigenous peoples’ rights to govern data about their communities, territories, and cultural practices — rights that may directly conflict with calls for open or centralised data access. Where citizen science projects involve Indigenous participants or knowledge, project designers should co-develop data governance agreements with relevant communities early, which may include tiered access controls or community-held repositories. Our recommendations referencing FAIR principles should be read with these considerations in mind.
- This recommendation responds to the findings from ‘Data collection and confidence’, ‘Recognition of volunteers’, and ‘Inclusion and access’ themes. There were many learnings about data collection, sharing and analysis. For data collection and sharing, online tools for citizen science need to work on multiple platforms and be inclusive and accessible, as exemplified by the ‘Sweaty and Stressed’ and ‘Marine Debris Initiative’ datasets. While four of the case studies we presented shared data according to the FAIR principles, five did not. This aligns with findings from other research that warns against projects where people donate their ‘unpaid work’ for data that may then be privately owned [9]. The use and promotion of large open data aggregators can ensure that data can continue to be accessible and used even once the citizen science initiative itself is no longer sustained. Important considerations for these data aggregators include clear quality controls, centralisation of the database, and the ability to flag incorrect records and filter data.

- 7. **Improve public involvement in all stages of research:** The public should be involved in all stages of science, from topic selection to project implementation and decision-making. This involvement should be reported transparently. This can be achieved by implementing inclusive involvement strategies with sufficient resourcing, including funding and staff time. All grant funding for citizen science by the government should require demonstration of how the public can be involved at each stage of the project. To ensure citizen science is inclusive, grant funding should provide specific funding to pay people for their time for being involved and ensure taxation practices do not inhibit involvement. This recommendation arises from the finding that 45% of participants felt only partially involved in project tasks. From these themes we identified exclusionary power dynamics and limited agency for participants.
- 8. **Expand training opportunities**: University-led and community-driven training, including formal credentials, could be offered to support both professional researchers and citizen scientists. Training and support networks should be established, including creating ‘communities of practice’ for early- to mid-career researchers who are initiating, leading or working on citizen science projects. This recommendation is informed by findings from the themes ‘Individual capability and training’ and ‘Data collection and confidence’, which indicate that insufficient training can constrain both the quality of engagement and the robustness of citizen science data. The C4RE Camps demonstrate how expert-led training passes on knowledge and builds wider community capacity. As citizen science increasingly incorporates new technologies, including data platforms and AI, training requirements expand. Training needs to extend beyond technical skills to include data interpretation, ethical practice, and responsible data management. In this way, confidence in data quality and trust in research processes can be maintained. Addressing these training needs can help build capacity on both sides of the research relationship and support sustainable participation and career pathways for participants [9,39].
- 9. **Encourage transparent and ethical research partnerships**: There is a need to improve ways for the public to be involved in setting priorities for both non-government and government funded research. Findings across the themes ‘Inclusion and access’, ‘Funding’, and ‘Project support and capacity’ indicate that opportunities for public involvement in decision-making are often constrained by structural factors. These include limited governance capacity and short-term or inflexible funding arrangements. These constraints reflect systemic barriers rather than an absence of public interest in participation. Transparent collaborations between industry, government, and academia can help address these constraints by clarifying roles, responsibilities, and decision-making processes. Such collaborations can also improve the availability of information about how well stakeholders align with the values and principles of citizen science and ethical research conduct. This type of partnership, which emphasises transparency, aligns with established guidance on opening science to society and strengthening ethical, participatory research governance [9,22].
- 10. **Improve consistency and terminology:** Relevant governments and international organisations (such as the United Nations, World Health Organization and the Citizen Science Global Partnership) should lead a process to ensure there is consistent terminology describing citizen science involvement and its underlying values and principles across all disciplines (e.g., health, environment and education) in multiple languages. Terminological consistency supports cross-sector recognition and integration of citizen science into policy and funding frameworks [3,6]. Calls in the literature for improved consistency and terminology [8,40] are supported by this recommendation. This recommendation came from the qualitative data across the themes ‘Attitudes’ and ‘Educational institutions’ that highlighted that inconsistent language can hinder funding eligibility and methodological legitimacy.

**Fig 4:**
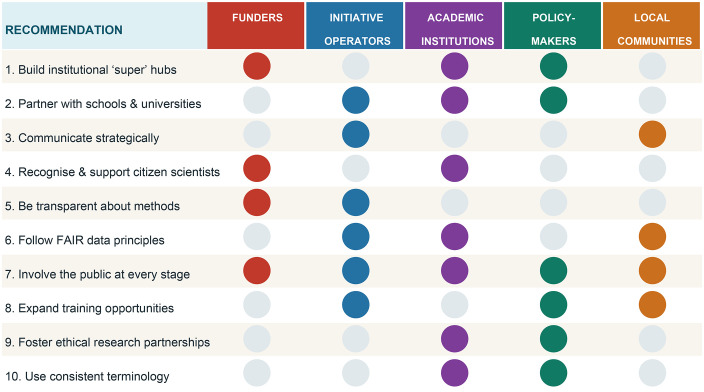
The recommendations that are most relevant and actionable to each major stakeholder group involved in citizen science initiatives.

Fig 4 below provides an overview of these recommendations, with each recommendation mapped to the most relevant stakeholders who should act on them: funders, citizen science initiative operators, academic institutions, policy makers, and local communities. A detailed overview of the recommendations along with key information about the paper is provided in the supplementary material as S1 Fig.

### Considerations for broader societal context

#### Long-term funding required.

Involving the public through citizen science is central to many formats of research, yet one respondent commented that “there is a lack of infrastructure to support making it easier for Early and Mid-Career Researchers (EMCRs) and community to connect”. This highlights the need for improved long-term resourcing (including funding, staff time and infrastructure) from governments, universities and other stakeholders. Such long-term resourcing can improve impacts of citizen science by providing sufficient central administration and data infrastructure, along with capacity building and training for both the wider public and people leading citizen science (for example at educational institutes). Given uncertainty in global funding for science, aid and conservation, citizen science should also explore diverse funding sources – while maintaining transparency on competing and conflicting interests. The use of centralised hubs that support decentralised science may be an efficient mode for utilising novel long-term funding sources.

#### Funding for inclusive and efficient ways of working.

Our results indicate that while people are more willing to complete unpaid tasks in some areas of science (such as data collection), there are clear areas for which citizen science projects need to be directly funded and resourced. Responses to our survey suggest a hybrid model, where some people volunteer and others are paid to complete specialised tasks, such as project management and navigating ethics processes. Such paid tasks could be efficiently centralised by organisations, with expertise in project management and ethics shared amongst multiple citizen science projects. Responses highlighted that lack of centralised support structures, including project management positions, were a challenge for operating successful citizen science. Importantly, while projects may plan to rely on volunteers, certain ways of working may exclude some people from projects where people are not remunerated. This may include those unable to afford to volunteer their time (including those with caring responsibilities) or those without access to certain technologies. Co-designing a project with multiple stakeholders can help ensure it is more inclusive, aligned with people’s expectations, culturally safe, and realistic. Co-designing involvement plans in this way can help ensure adequate support is available for people to become involved. Important factors may involve budgets for payment and expenses, technical support, or if appropriate, emotional support.

We also acknowledge that our definition of ‘paid’ in this study is intentionally narrow and generally refers either to hourly financial compensation or to payment as part of a salaried position, such as administration staff employed by institutions. We note that there are other reward mechanisms such as honorariums and non-financial rewards that are more appropriate for some participants, and which were identified in survey responses under the theme ‘recognition of volunteers’. Such non-financial compensation may include scientific library access, co-authorship, education, or conference attendance. People should be involved in deciding the kind of compensation or rewards that are most appropriate for them.

#### Legal and ethical guidance on the use of artificial intelligence is urgently required.

Technologies, such as AI, are making new ways of working possible as the cost, accessibility and usability of such technologies improve. AI will inevitably play an increasingly essential role in future citizen science by enlarging the scale of data that can be analysed, and supporting data collection, analysis, dissemination and translation [66]. However, the ethical use and environmental impact of such tools and technologies often remain unclear, and further legal and ethical guidance from both governments and other stakeholders is urgently required [67,68]. Additionally, as with all methodologies, improved reporting on the use of AI needs to be transparently shared, including the underlying code and training data.

#### Improved data about political and other influences on science is required.

Politicisation of scientific findings is increasing, as are misinformation and disinformation that contribute to the ongoing “infodemic” [69]. As citizen science increasingly contributes to large-scale data collection and public involvement in research, safeguards from the politicisation of citizen-led research are needed [70]. Areas of research including climate science, public health, drug development, and mental health remain politicised. Improved transparency of data will aid public understanding of where policy is influenced by evidence, ideology or economics. For example, multiple political factors are negatively impacting citizen science initiatives on climate change, including the ability of citizens and institutions to collect, share and interpret essential climate data [71–74]. Globally, there is increasing political and corporate interference in climate science. This has included the funding of misinformation via fossil fuel companies who also sponsor government policy [75]. In some countries, such as the USA, China, and Australia, government policies restrict access to environmental information [74,76]. The defunding of climate institutions, shifts in public communication under political influence, and subsequent self-censorship of researchers all negatively shape citizen science impacts [71,77]. Globally, citizen science can continue to be a force to collect, share and inspire action, but its impacts decrease following politicisation of science. Improved transparency about political and financial influences on citizen science can strengthen trust in both the research and the institutions involved.

#### Evidence-informed methods of research dissemination and translation are needed to uphold democracies.

A key benefit of citizen science is the direct involvement of communities and the public in science, providing awareness and direction for scientific research that reflects the interests of the public. Citizens within democracies should review and co-create the ‘enabling conditions’ for science, including clear definitions and distinctions of what is ‘dissemination’ and ‘translation’, and what is ‘protest’ [78]. In 2022, the United Nations Secretary-General stated “climate activists are sometimes depicted as dangerous radicals. But, the truly dangerous radicals are the countries that are increasing the production of fossil fuels” [79]. In this context, the scientific method must objectively catalogue the methods of disseminating and translating research, including protests, alongside cataloguing the vested interests of people working for industries and other stakeholders, and their influence on government policies. Tools such as STARDIT can be used to map such interests in a transparent and collaborative way [45].

For people to be involved in citizen science, they require certain ‘enabling conditions’, which include transparency and personal safety. Consideration should be given to the duty of care owed by organisations conducting citizen science, particularly in relation to the personal safety of participants. This includes safeguarding individuals from potential harassment that may arise during data collection, dissemination, or translation of research. Further clarity is required on where the duty of care is situated in citizen science, and who might share it, including legislative, executive, or judicial bodies. For example, lawmakers could either reframe public events to share scientific data as a legal form of scientific research translation and dissemination, or as ‘illegal protest’. The risk of strategic lawsuits against public participation (SLAPPs) is also significant, and may impact people’s willingness to initiate, participate in or disseminate and translate results from citizen science [80]. Legislation is required world-wide to dissuade such methods.

By analysing various methods of research translation and dissemination (through proper reporting and evaluation), we can explore evidence-informed approaches for translating scientific knowledge into action to prevent crises such as irreversible climate change and mass-extinction, and better protect those involved in translating findings from citizen science. Better reporting of such data will strengthen our collective ability to learn about safe and successful methods of dissemination and translation.

### Study Limitations

The survey responses were analysed qualitatively because the sample size of 46 was not large enough to make statistical inferences. Most respondents were Australian, and although the results primarily reflect the Australian context and perspectives, the findings and learnings are considered broadly generalisable. There was also an unequal representation of fields. For example, ecology was heavily represented while some fields were underrepresented, such as health (despite its significant share of governmental budget spending), or absent, such as climate and weather projects. The survey likely targeted individuals already engaged in citizen science, which introduced a bias toward those who view it positively. Perspectives from stakeholders with competing interests who might be less supportive of citizen science may be underrepresented. While responses included a range of stakeholders, the sample was skewed toward academia. This overrepresentation may have narrowed the range of perspectives, which in turn may have affected emerging themes and potentially omitted key issues important to the public. While the sample size is relatively small, the co-authors agreed that the survey responses, including 16,247 words in open text fields, as well as the analysis of identified citizen science projects enabled the identification of key barriers and opportunities for citizen science and added valuable contributions to the field.

The generalisability of the 10 recommendations presented in this article should be interpreted with caution in contexts that differ substantially from the study sample. Given the predominantly Australian, academic, and ecology-oriented composition of respondents, several important contextual limitations warrant acknowledgement.

In Low- and Middle-Income Countries (LMICs), the structural preconditions assumed by several recommendations (including long-term government funding, centralised university infrastructure, and access to digital platforms) may not be readily available. Additionally, power dynamics between international funders or institutions and local communities can complicate the co-production principles central to our recommendations. Recommendations related to institutional ‘super hubs’, formal credentialing, and mandated FAIR data sharing may require significant adaptation to be feasible and equitable in lower-resource contexts. Future research should explore how these recommendations translate to citizen science initiatives in LMICs, where community-led models and existing local knowledge systems may offer alternative pathways to the ones we have identified. Nonetheless, citizen science is inherently a global endeavour, and these recommendations should not be read as exclusively relevant to high-income, English-speaking contexts. Higher-income countries, institutions and funders have both the opportunity and responsibility to support citizen science in LMICs.

In clinical and health research contexts, the degree of public involvement and data openness our recommendations advocate may require adaptation to ensure appropriate ethical oversight. Further research is required to ensure a balance between public interest and commercial interests (including those of pharmaceutical sponsors or private research funders) which encourages transparency and the co-production methods that are central to this articles’ recommendations.

#### Perceived gaps in the data.

As part of the data analysis process, the co-authors discussed the results and agreed areas or themes where they had expected to see more data or responses. The co-authors expected ‘open data’ and ‘data sharing’ to be more prominent. Similarly notable areas where no examples of best practice were shared were ‘weather’, ‘climate’ and ‘air pollution’, despite these being significant areas for citizen science globally [33]. The environmental impact of citizen science itself was a theme that was also absent, along with any recommendations for offset practices [81].

### Knowledge gaps and opportunities for further research

As part of this research, we have identified two key knowledge gaps and opportunities for further research. Firstly, while we suggest that many of the themes and challenges identified as part of this research are reflective of wider patterns across the world, there are also likely large differences across social, economic, technological, political and cultural contexts. Secondly, while the citizen science experts surveyed as part of this research hold key insights into how the impacts of citizen science can be improved, we recognize that other stakeholder groupings – including policymakers, people working for industry, and people from marginalised communities – hold important perspectives for strengthening their own involvement in citizen science projects and improving the overall outcomes of citizen science initiatives.

Further research could explore both gaps by extending the approach applied in this research to new geographic contexts and stakeholder groupings, including policymakers, industry actors, and marginalised communities. A key element of this work could involve identifying best practices that can be scaled, replicated, and used to strengthen international collaboration and better leverage the transformative potential of citizen science. Future work could also adopt more structured and transparent approaches to planning and evaluation, including assessing impact, feasibility, costs, and time horizons, alongside developing shared indicators and monitoring frameworks aligned with FAIR data principles and using STARDIT reporting to support comparability and accountability across initiatives.

## Conclusion

Citizen science has the potential to play a key role in generating and applying scientific knowledge, and supporting the environmental and social action necessary for addressing the complex and multifaceted global challenges in the 21^st^ century. The inclusion of people and communities in shaping the future of science in multiple disciplines (including health, environment and education) has become increasingly important as the climate changes and more ecosystems collapse, threatening the health and survival of multiple lifeforms. This study provides a co-produced analysis of structural and operational barriers that currently limit the potential of citizen science, alongside ten actionable recommendations for strengthening citizen science across disciplines. Our findings demonstrate that methodological robustness, participant recognition, institutional infrastructure, and funding stability are interdependent factors shaping both data credibility and sustained public involvement.

For policymakers and funders, realising the full societal value of citizen science requires sustained institutional support, FAIR-aligned data governance, transparent recognition systems, and expanded training pathways. Strengthening citizen science will require better project design and systemic reform that embeds participatory research within national and international research infrastructure, using evidence-informed methods.

Rather than viewing citizen science as an auxiliary data-collection method, the evidence presented here supports a paradigm shift. It reframes citizen science as an institutional ecosystem, requiring long-term investment, governance clarity, and ethical transparency. Structural instability, particularly short-term funding cycles and inconsistent recognition practices, undermines participant retention and data continuity, even in otherwise successful initiatives. To foster impactful citizen science initiatives, providing opportunities, spaces and funding enables researchers to build experiences and critical long-term connections necessary to improve citizen science. Improving systematic documentation and reporting of methods, impacts and outcomes of involving people in science (such as using STARDIT) will enable a more evidence-informed approach to improving inclusivity and resilience in citizen science. Using such evidence-informed approaches to improve citizen science through systematic changes will help ensure improved benefits to society, global health, and the very ecosystems on which all life depends.

## Supporting information

S1 File Glossary. (DOCX)

S1 Table Overview of characteristics of respondents. (DOCX)

S2 Table The themes and keywords associated with each theme used for the thematic analysis heatmap. (DOCX)

S1 Fig Recommendations and key information about the paper. (PNG)
