# Supplementary material for: 10 recommendations for strengthening citizen science for improved societal and ecological outcomes: A co-produced analysis of challenges and opportunities in the 21st century
Source: PLoS One. 2026 Jul 1;21(7):e0331161. doi: 10.1371/journal.pone.0331161 (PMC13322523; doi:10.1371/journal.pone.0331161)
Supplement: S1 File — (DOCX) [file pone.0331161.s001.docx]

## Glossary

This glossary is not intended as definitive definitions of the listed terms, but rather to convey how they have been used in this paper.

| **Term** | **Definition** |
| --- | --- |
| Artificial intelligence tools | The use of tools which encompasses the use of intelligent machines and ‘machine learning’ to achieve specific tasks |
| Citizen science | Public participation, involvement and collaboration in scientific research with the aim to increase scientific knowledge |
| Citizen scientists | People involved, but volunteering, not being paid |
| Educational institutions | Schools, universities and charities |
| Engagement | When information and knowledge about research is shared with the public to better inform them on why, how, where, and by whom research is conducted |
| Involvement / involving | Meaning when people are actively involved in working with professional researchers and research organisations to help shape research priorities, design, conduct, dissemination, translation and evaluation of research |
| Non-professional scientists | Someone who is not employed as a professional scientist but actively participates in scientific research, often in collaboration with professional scientists |
| Professional researchers | People paid to do research |
| Professional staff | People paid to support with a project |
| Retention | People staying involved in a project |
