# Supplementary material for: 10 recommendations for strengthening citizen science for improved societal and ecological outcomes: A co-produced analysis of challenges and opportunities in the 21st century
Source: PLoS One. 2026 Jul 1;21(7):e0331161. doi: 10.1371/journal.pone.0331161 (PMC13322523; doi:10.1371/journal.pone.0331161)
Supplement: S1 Table — (DOCX) [file pone.0331161.s002.docx]

## Table S1: Overview of characteristics of respondents

| **Overview of respondent characteristics** | **Percent (number)** |
| --- | --- |
| I live in Australia | 80% (37) |
| I consider myself to be an 'academic' and have experience of academic writing | 76% (35) |
| I am a professional researcher paid to do research | 65% (30) |
| I have experience of being a volunteer researcher who contributes to citizen science | 65% (30) |
| I am an early to mid-career researcher | 50% (23) |
| I am 'neurodivergent' | 20% (9) |
| I am an Aboriginal or Torres Strait Islander person, or Indigenous person | 4% (2) |

## 
