# Supplementary material for: 10 recommendations for strengthening citizen science for improved societal and ecological outcomes: A co-produced analysis of challenges and opportunities in the 21st century
Source: PLoS One. 2026 Jul 1;21(7):e0331161. doi: 10.1371/journal.pone.0331161 (PMC13322523; doi:10.1371/journal.pone.0331161)
Supplement: S2 Table — (DOCX) [file pone.0331161.s003.docx]

## Table S2: Themes and keywords in thematic analysis heatmap

| **Theme** | **Keywords** |
| --- | --- |
| Recruitment and awareness | Recruitment, infrastructure, connect, community, marketing, attract, retain, outreach, advertise, promote, visibility, communication |
| Involvement and retention | Involvement, participation, turnaround, active, casual, ongoing, accessible, intuitive, skill, collection, engage, retention |
| Attitudes | Prestigious, recognition, consumer, engagement, perception, academic, science, feeling, understanding |
| Schools and education | School, education, certificate, training, authentic, learning, teach, student, classroom, curriculum |
| Inclusion and access | Power, structure, space, pathway, ethical, indigenous, inclusive, respectful, diversity, equity, barrier |
| Data collection and confidence | Data, collection, confidence, database, protocol, metadata, validation, reliability, valid, quality, inform, collection |
| Recognition of volunteers | Recognition, volunteer, reward, certificate, newsletter, access, acknowledge, contribution, value, exchange, insight |
| Individual capability and training | Individual, capability, training, skill, time, engage, diverse, integrity, peer-review, resource, stakeholder |
| Project support and capacity | Support, capacity, website, database, resource, promote, hub, enabling, self-managed, infrastructure, platform |
